# Evaluating survey designs for targeting preventive chemotherapy against *Schistosoma haematobium* and *Schistosoma mansoni* across sub-Saharan Africa: a geostatistical analysis and modelling study

**DOI:** 10.1186/s13071-020-04413-7

**Published:** 2020-11-18

**Authors:** Kimberly M Fornace, Claudio Fronterrè, Fiona M. Fleming, Hope Simpson, Honorat Zoure, Maria Rebollo, Pauline Mwinzi, Penelope Vounatsou, Rachel L. Pullan

**Affiliations:** 1 Department of Disease Control, London School of Hygiene and Tropical Medicine, London, UK; 2 Centre for Health Informatics, Computing and Statistics (CHICAS), Lancaster University, Lancaster, UK; 3 Schistosomiasis Control Initiative, London, UK; 4 Expanded Special Project of the Elimination of Neglected Tropical Diseases (ESPEN), Africa Regional Office of the World Health Organisation, Brazzaville, Congo; 5 Swiss Tropical and Public Health Institute, Basel, Switzerland

## Abstract

**Background:**

Schistosomiasis control programmes primarily use school-based surveys to identify areas for mass drug administration of preventive chemotherapy. However, as the spatial distribution of schistosomiasis can be highly focal, transmission may not be detected by surveys implemented at districts or larger spatial units. Improved mapping strategies are required to accurately and cost-effectively target preventive chemotherapy to remaining foci across all possible spatial distributions of schistosomiasis.

**Methods:**

Here, we use geostatistical models to quantify the spatial heterogeneity of *Schistosoma haematobium* and *S. mansoni* across sub-Saharan Africa using the most comprehensive dataset available on school-based surveys. Applying this information to parameterise simulations, we assess the accuracy and cost of targeting alternative implementation unit sizes across the range of plausible schistosomiasis distributions. We evaluate the consequences of decisions based on survey designs implemented at district and subdistrict levels sampling different numbers of schools. Cost data were obtained from field surveys conducted across multiple countries and years, with cost effectiveness evaluated as the cost per correctly identified school.

**Results:**

Models identified marked differences in prevalence and spatial distributions between countries and species; however, results suggest implementing surveys at subdistrict level increase the accuracy of treatment classifications across most scenarios. While sampling intensively at the subdistrict level resulted in the highest classification accuracy, this sampling strategy resulted in the highest costs. Alternatively, sampling the same numbers of schools currently recommended at the district level but stratifying by subdistrict increased cost effectiveness.

**Conclusions:**

This study provides a new tool to evaluate schistosomiasis survey designs across a range of transmission settings. Results highlight the importance of considering spatial structure when designing sampling strategies, illustrating that a substantial proportion of children may be undertreated even when an implementation unit is correctly classified. Control programmes need to weigh the increased accuracy of more detailed mapping strategies against the survey costs and treatment priorities.
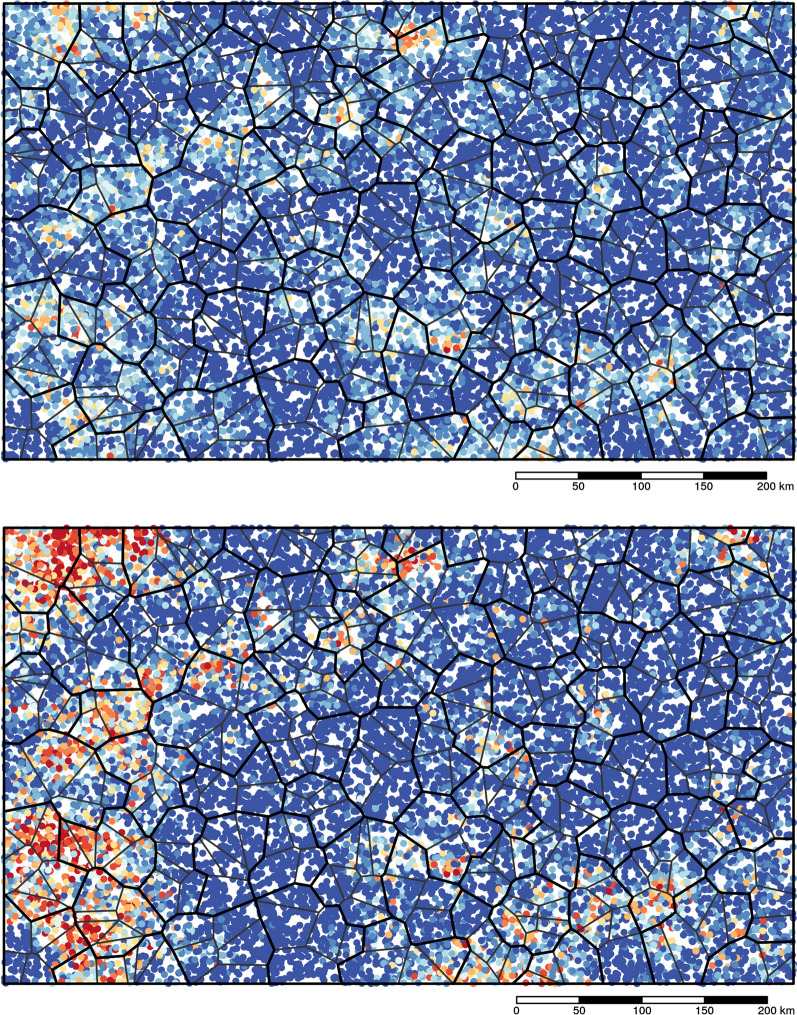

## Introduction

Schistosomiasis is a chronic parasitic disease with substantial public health impacts globally. The majority of the burden occurs in sub-Saharan Africa, with over 150 million individuals estimated to be infected with either *Schistosoma haematobium* or *S. mansoni* [1]. Determined by the distribution of and contact with freshwater snail habitats and access to clean water and sanitation, schistosomiasis transmission is highly focal, with disease risks varying markedly within small spatial areas [2, 3]. This spatial heterogeneity has become more pronounced as prevalence decreases due to highly successful control programmes [4]. New strategies are required to identify and target remaining foci of infection in order to achieve elimination goals, including re-evaluating the spatial units at which control programmes operate.

Preventive chemotherapy with praziquantel remains the cornerstone of schistosomiasis control programmes [4, 5]. Mass drug administration targets high risk groups living in endemic areas, including school aged children (SAC, ages 5–14 years), pregnant women and individuals with occupational risks [6]. Prior to treatment, epidemiological data on infection prevalence are required to identify high-risk communities within geographically suitable regions; prevalence is assessed, and treatment assigned at a spatial unit referred to as the implementation unit (IU). Although there is no standard definition for IUs, these are typically defined as districts or ecological zones consisting of multiple subdistricts [7]. Current World Health Organization (WHO) guidelines recommend purposefully selecting sites in high prevalence areas. In practice, most countries identify high prevalence regions and estimate prevalence using two-stage cluster-based school surveys, sampling 50 children per school for up to five schools per IU [6, 7]. This survey design is easily implemented and analysed, using simple random sampling from lists of schools per IU without requiring additional fine-scale spatial information on schistosomiasis distribution. Based on these surveys, preventive chemotherapy is administered every year for mapping units with over 50% prevalence, every two years if the prevalence is between 10–50% and every three years if prevalence is below 10% [6]. These guidelines are currently under review by a WHO working group; although no official guidelines have been finalised, forthcoming guidelines are likely to specify a single 10% threshold determining annual treatment [8]. As countries move towards elimination and schistosomiasis transmission becomes more focal, there is increasing recognition of the need to implement treatment policies at lower administrative levels [9]. Previous studies have demonstrated a substantial proportion of schools are misclassified and incorrectly treated, due to variation within districts [10]. The rollout of these new guidelines makes this an opportune time to evaluate potential sampling and treatment strategies implemented at district or subdistrict levels [11].

Determining an optimal sampling strategy requires balancing trade-offs between accuracy and cost to determine where preventive chemotherapy should be targeted. Simulations are frequently used to assess how alternative sampling strategies can improve estimates of prevalence and maximise cost efficiency by more accurately targeting preventive chemotherapy [12–15]. Using geostatistical simulations of baseline prevalence estimated from existing survey data, effects of different sampling strategies can be evaluated against known parameters generated from spatially realistic simulated datasets. This allows assessment of a wide range of sampling designs across different prevalence distributions. For schistosomiasis, this approach has been used to explore the accuracy and cost effectiveness of varying the number of schools and number of children sampled under current two-stage cluster design surveys [16]. Additionally, a previous study explored the performance of two sampling strategies for classifying school-level schistosomiasis prevalence: lot quality assurance sampling (LQAS), sampling small numbers of children from all schools within a given area, and model-based geostatistical approaches, using spatial modelling approaches to predict prevalence based on a small number of sampled schools [12].

Here, we use geostatistical simulations to evaluate potential survey sampling strategies across a range of plausible spatial distributions of *S. haematobium* and *S. mansoni* prevalence. We compare two-stage cluster survey designs implemented at district and subdistrict levels sampling different numbers of schools. We aim to (i) quantify the spatial heterogeneity of schistosomiasis across sub-Saharan Africa; (ii) evaluate the accuracy of survey designs across this range of parameters; and (iii) assess the cost implications of survey designs. Together, this allows identification of optimal survey designs across spatially heterogenous distributions of disease.

## Methods

To evaluate sampling strategies across all possible distributions of schistosomiasis in SSA, we first fitted country and species-specific geostatistical models to characterise the spatial heterogeneity of schistosomiasis. These models were then used to parameterise simulations in a representative, hypothetical country in SSA, capturing the full range of possible transmission scenarios. Alternative sampling strategies were assessed based on the overall accuracy, numbers of schools correctly and incorrectly treated as well as estimated cost implications.

### Country-level geostatistical analysis

To estimate the spatial distribution of schistosomiasis, we obtained data on schistosomiasis surveys from the WHO Expanded Special Project for Elimination of Neglected Tropical Diseases (ESPEN) portal [17]. This represents the largest and most geographically comprehensive database of schistosomiasis surveys. We assembled data for all georeferenced school-based surveys of school-age children in SSA. To exclude survey points with inaccurate spatial data, we removed all surveys with duplicate locations reported within the same year and surveys with coordinates reported outside the district or administrative unit of the named school/site. We additionally excluded survey points reporting only a prevalence rather than the numbers of children sampled and detected positive. We then excluded schools reporting over 100 children sampled as these were not representative of typical survey methodology and potentially reflected aggregated data. In line with current WHO guidelines, we defined *S. haematobium* using urine filtration and *S. mansoni* infections diagnosed using Kato-Katz techniques. For countries with repeated survey data reported for multiple years, we included only the most recent survey. Countries with less than 50 survey points were excluded from further analysis.

From this dataset, we fitted binomial geostatistical models to schistosomiasis data. Models were fitted separately for each country and species, with no additional covariates included. The number of individuals, *Y*_*i*_, who tested positive for schistosomiasis out of the total number of individuals examined, *n*_*i*_*,* at location *x*_*i*_ was considered as the realisation of a binomial random variable *Y*_*i*_ ~ Binomial(*n*_*i*_*, p*(*x*_*i*_)), with *p*(*x*_*i*_) modelled as:

1 $$\mathrm{log}\left\{\frac{p\left({x}_{i}\right)}{1- \left(p\left({x}_{i}\right)\right)}\right\} = \mu +S\left({x}_{i}\right)+ {Z}_{i}$$

where μ is the intercept, $S\left({{x}_{i}}\right)$ is a zero mean Gaussian process with variance *σ*^*2*^ and an exponential correlation function given by *ρ*(*k; φ*) = exp{-*k/ φ*} where *φ* > 0 is a scale parameter that controls the extent of spatial correlation and *k* is the distance between two sampling locations; and ${Z}_{i}$ is a set of independent zero-mean Gaussian variables with variance *τ*^*2*^. Models were fitted using Monte Carlo maximum likelihood estimation implemented in R [18].

### Schistosomiasis prevalence simulations

As we wanted to evaluate survey strategies for all possible schistosomiasis distributions, we chose to simulate prevalence within a hypothetical representative country rather than using data for single countries. We assigned this simulated country an area representing mean country area of all countries in SSA (km^2^). We used the median ratio of numbers of district level IUs to country area in SSA to determine the number of districts. Estimating five subdistricts per IU on average, we randomly assigned districts and subdistricts, with all districts comprised of 5 subdistricts. The final country had district and subdistrict sizes comparable to the average geographical sizes observed across SSA countries. As our simulated country had a similar area to Uganda, we distributed schools based on the density of schools within Uganda, assuming 500 SAC per school and at least 5 schools per district, leading to a total estimate of 15,000 schools countrywide (Additional file 1: Figure S2).

To capture heterogeneity of the spatial variability and spatial extent of schistosomiasis across SSA, we combined parameters from all geostatistical models to generate gold-standard prevalence distributions. For each species, we defined schistosomiasis using all possible combinations of the median, 25th and 75th percentile of country level parameters fitted from geostatistical models, across deciles of mean prevalence. This allowed simulations of prevalence surfaces capturing all possible scenarios with the full range of prevalence levels, spatial variances and scales. For each combination of model parameters, we conducted 100 unconditional simulations of the number of SAC positive in all schools within the hypothetical country.

### Evaluation of alternate survey designs

Using these simulations, we assessed different survey designs. These included: (i) sampling 5 randomly selected schools per district, with the IU defined as a district (existing sampling strategy); (ii) sampling 5 randomly selected schools per subdistrict, using subdistrict as the IU; and (iii) sampling 1 randomly selected school per subdistrict, with IU defined as a subdistrict. For all sampling strategies, we sampled 50 randomly selected SAC per selected school. As per current guidelines, we used the mean prevalence per IU to determine whether the school was above or below a threshold. To evaluate survey designs, we compared the survey classification of the IU to the gold standard classification calculated from the mean prevalence of all schools within the IU. Survey designs were assessed based on overall accuracy of treatment classifications (at the level of the IU) as well as the proportions of schools over or undertreated (given school-level prevalence) using the resulting assigned IU treatment classification. We additionally compared the survey accuracy, with parameters defining the spatial distribution of schistosomiasis, to determine how survey performance varied across transmission settings.

### Cost analysis

As more intensive sampling may give more accurate results but be prohibitively expensive, we additionally assessed the cost of all survey designs. Survey design costs were estimated using the ingredients method using capital resources data obtained from school-based mapping surveys [16]. We considered only financial costs and excluded expenditures related to general programme operating costs or costs borne by the beneficiaries. Expenditures related were extracted for five annual programmatic surveys, including surveys conducted in 2016–2017 in Malawi and Uganda and surveys conducted in 2017–2018 in Tanzania, Malawi and Uganda (SCI, financial expenditure records). We calculated the mean costs per school surveyed separately for each of the five available surveys, using the median cost from all survey data to evaluate cost effectiveness. Consumable item costs were calculated based on the quantity used for each diagnostic method and number of children surveyed, assuming 10% wastage. We defined capital items as items with a typical life expectancy of over one year; the costs of these items were annuitized based on the useful life expectancy in years.

We assumed an average of one school per day would be visited by the survey team, including a half day to register children and collect samples and a half day of sample processing. Based on reported survey activities, teams included one driver, one team leader, one district officer and one central officer with three technicians would be required to sample 50 children. Per diems for sample teams were estimated based on reported country specific expenditures. We calculated mean costs per school based on reported district level fuel costs and numbers of schools covered, assuming vehicle maintenance was conducted once during the survey period. No capital costs for vehicle purchase were included. All costs were converted into US dollars (USD) using the Consumer Price Index and current exchange rates. Cost effectiveness was evaluated based on the cost per school assigned to the correct treatment category using the median survey cost per school. This definition of cost effectiveness prioritises classification accuracy, weighting schools not requiring treatment and requiring treatment equally. However, alternatively, control programmes may prioritise ensuring all schools above the prevalence threshold receive treatment. To address this priority, we additionally evaluated the cost per school requiring treatment which was adequately treated.

## Results

**Fig. 1:**
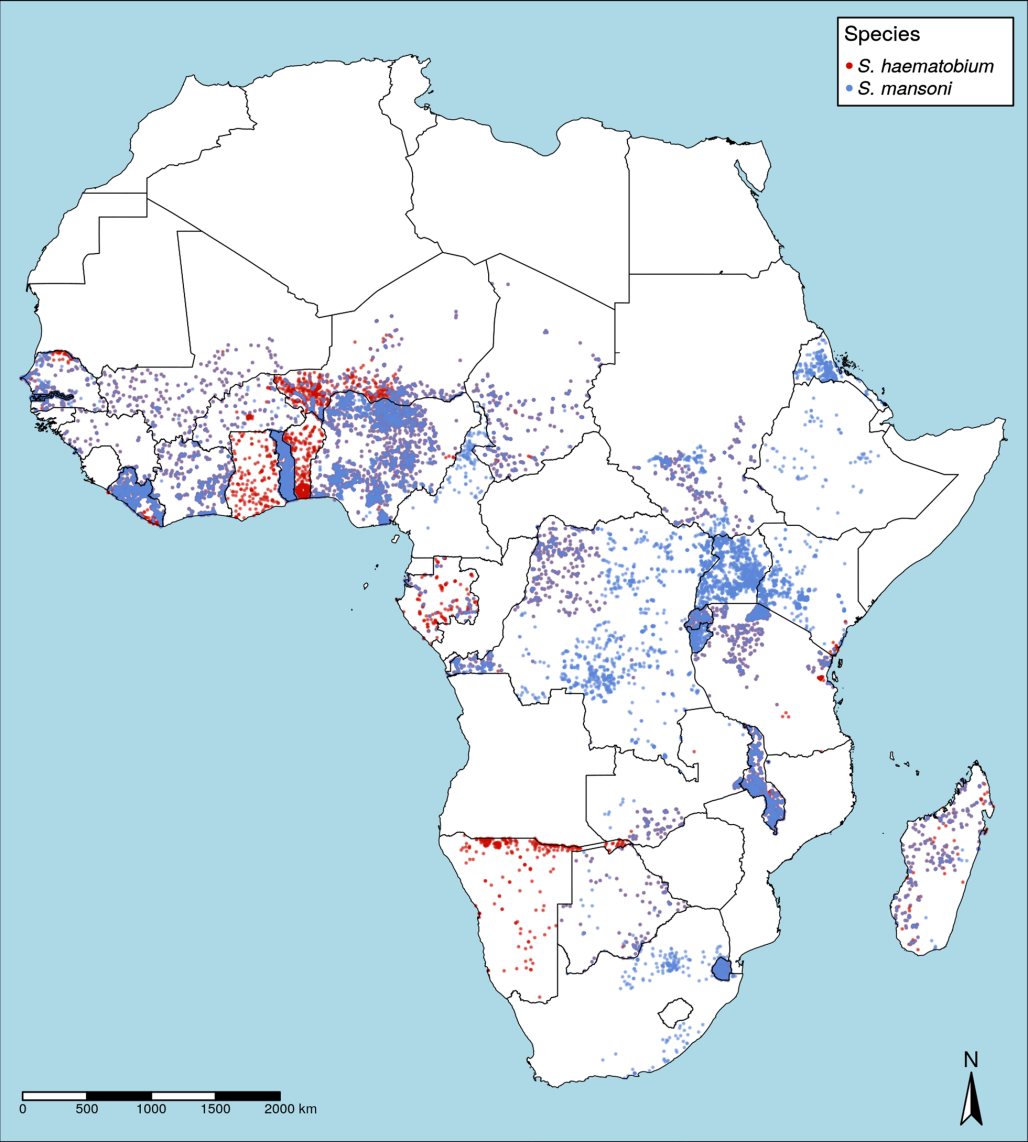
Distribution of school-based surveys included in the analysis

**Fig. 2:**
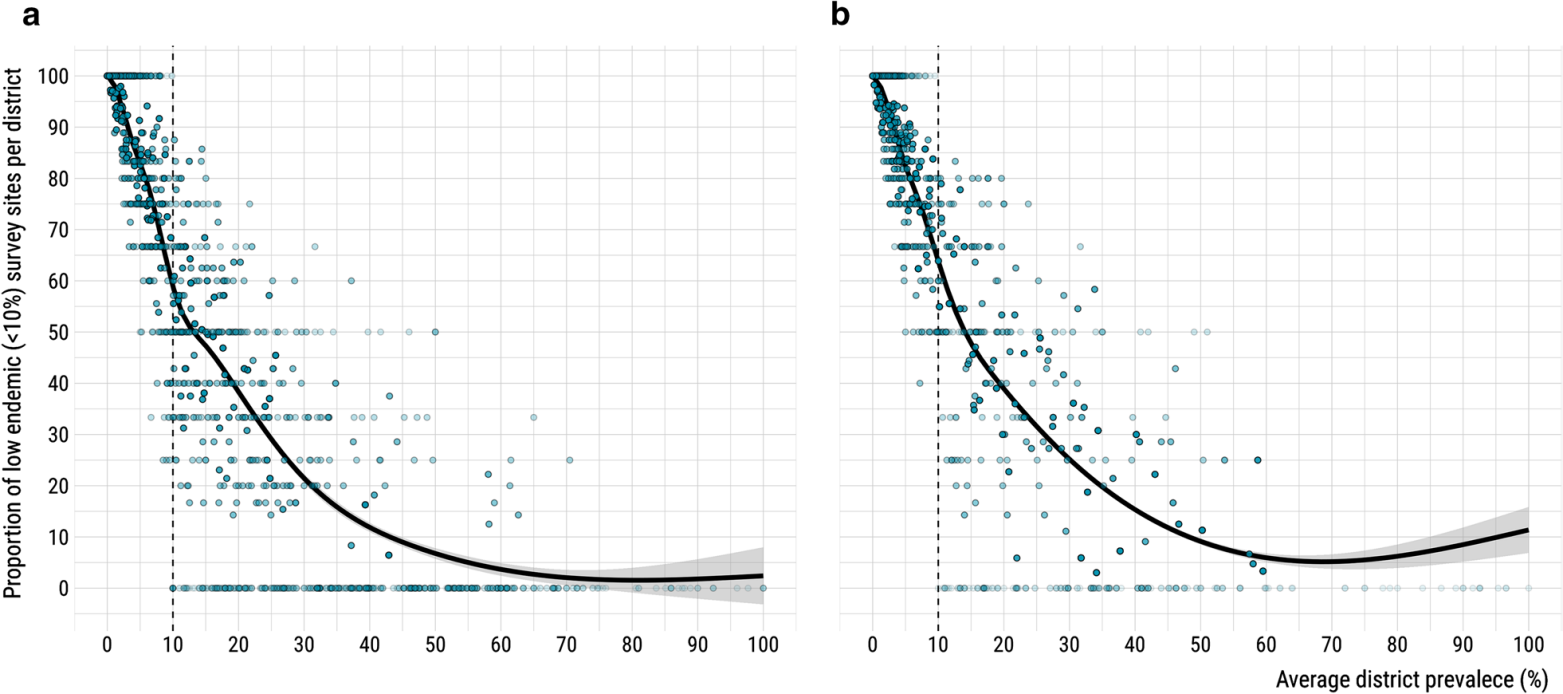
Average district prevalence and distribution of included schools with prevalence below 10% for all surveys included for *S. haematobium* (**a**) and *S. mansoni* (**b**)

**Fig. 3:**
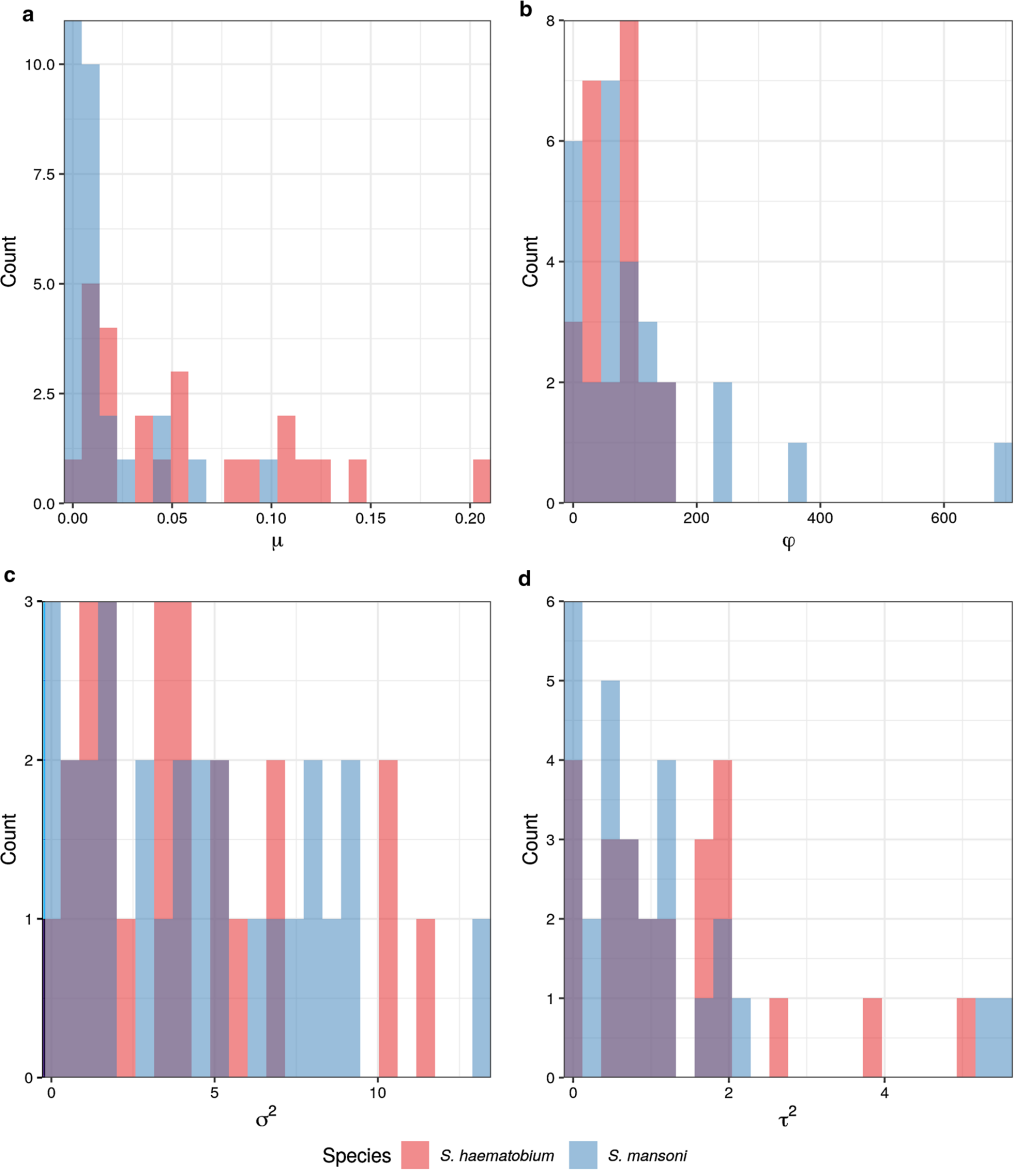
Monte Carlo maximum likelihood estimates for geostatistical model parameters by species

We fitted geostatistical models for *S. haematobium* prevalence in 24 countries and *S. mansoni* in 28 countries across SSA (Fig. 1). This dataset included results from 23,722 school-based surveys, with the numbers of schools sampled per country ranged from 64 to 1838 for *S. haematobium* and 64 to 2230 for *S. mansoni* (Additional file 1: Figure S1). For *S. haematobium*, the median school-level prevalence was 0.02 (interquartile range, IQR: 0–0.12), with 3039 of the 10,316 schools sampled (29.5%) reporting a prevalence of 10% or higher (Fig. 2). Median prevalence of *S. mansoni* was 0.00 (IQR 0–0.04) with only 2529 of 13,406 schools sampled (18.9%) exceeding the 10% threshold. For both species, the median number of children sampled per school was 50 (IQR: 30–50). Spatial patterns of prevalence varied markedly between countries and species surveyed, with *φ* ranging from 0.07 to 146.44 km (mean: 73.30 km) for *S. haematobium* and 0.0048 to 696.13 km (mean: 109.23 km). Similarly, both spatial variance (*τ*^*2*^) and nonspatial variance (*σ*^*2*^) differed by country and species (Fig. 3, Additional file 1: Table S1).

**Fig. 4:**
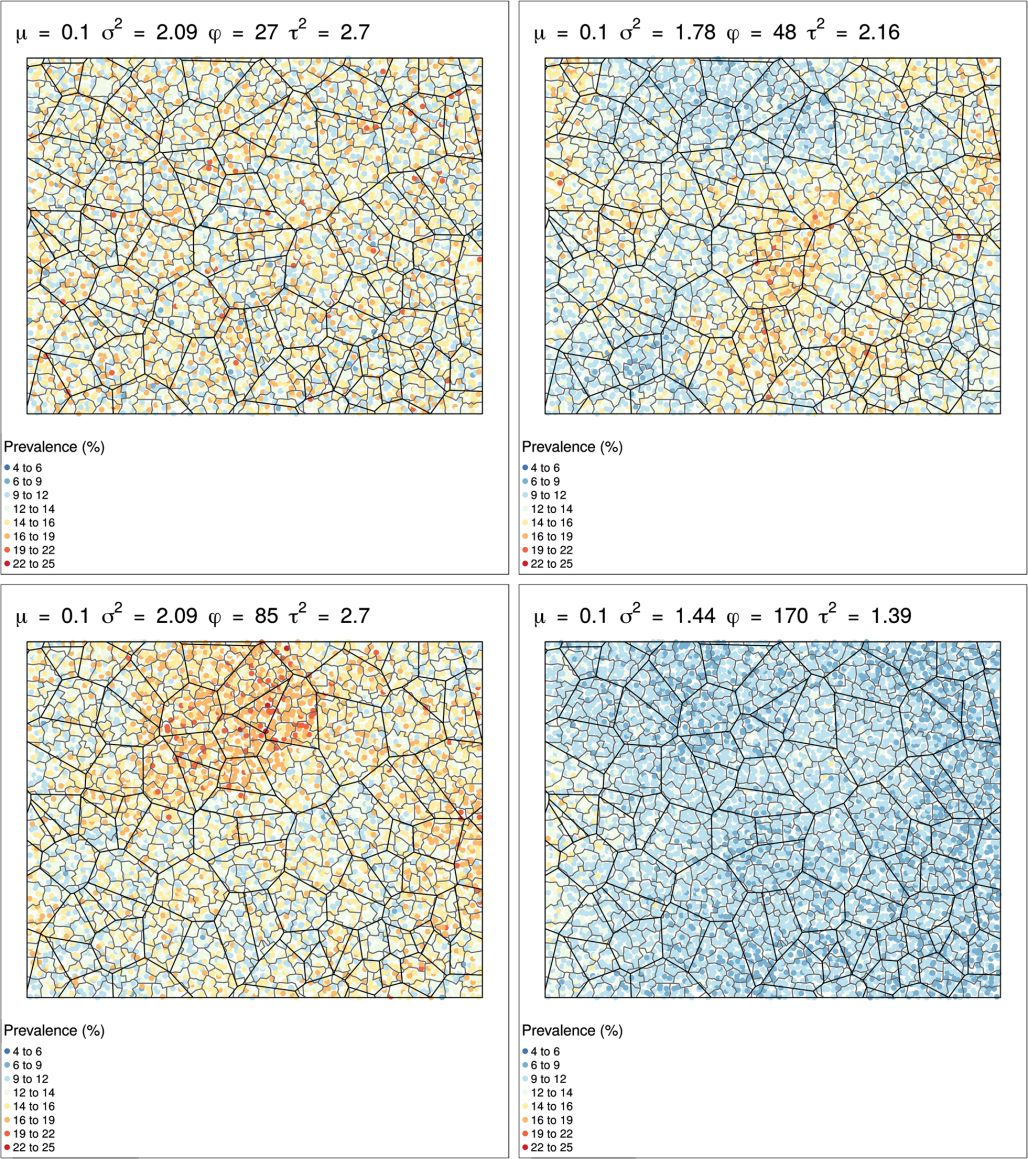
Examples of effects of model parameters on the spatial distribution of prevalence assuming a constant mean prevalence of 0.1 across all scenarios

Using these models, we generated prevalence surfaces across our simulated country, representing plausible spatial distributions of infections. From country-specific geostatistical models, we used the median, 25th and 75th quartiles for all possible combinations of model parameters *φ, τ*^*2*^ and *σ*^*2*^ with *µ* ranging from 0.0005 to 0.207 (Table 1). This generated 297 unique scenarios each for *S. haematobium* and *S. mansoni*. These scenarios represented all possible distributions of prevalence with different levels of spatial and nonspatial variance and ranges of spatial correlation across a simulated country (Fig. 4).

**Table 1:** Model parameters used for simulations

Parameter	*S. haematobium*	*S. mansoni*
*µ*	0.004, 0.008, 0.012, 0.017, 0.024, 0.040, 0.054, 0.079, 0.110, 0.125, 0.207	0.0005, 0.0013, 0.0026, 0.0038, 0.0048, 0.0058, 0.0068, 0.0077, 0.0175, 0.0423, 0.0991
*φ*	30.58, 73.30, 89.71	41.74, 61.64, 114.01
*τ*^*2*^	0.558, 0.977, 1.826	0.312, 0.655, 1.266
*σ*^*2*^	1.458, 3.642, 5.298	1.662, 4.056, 7.058

**Fig. 5:**
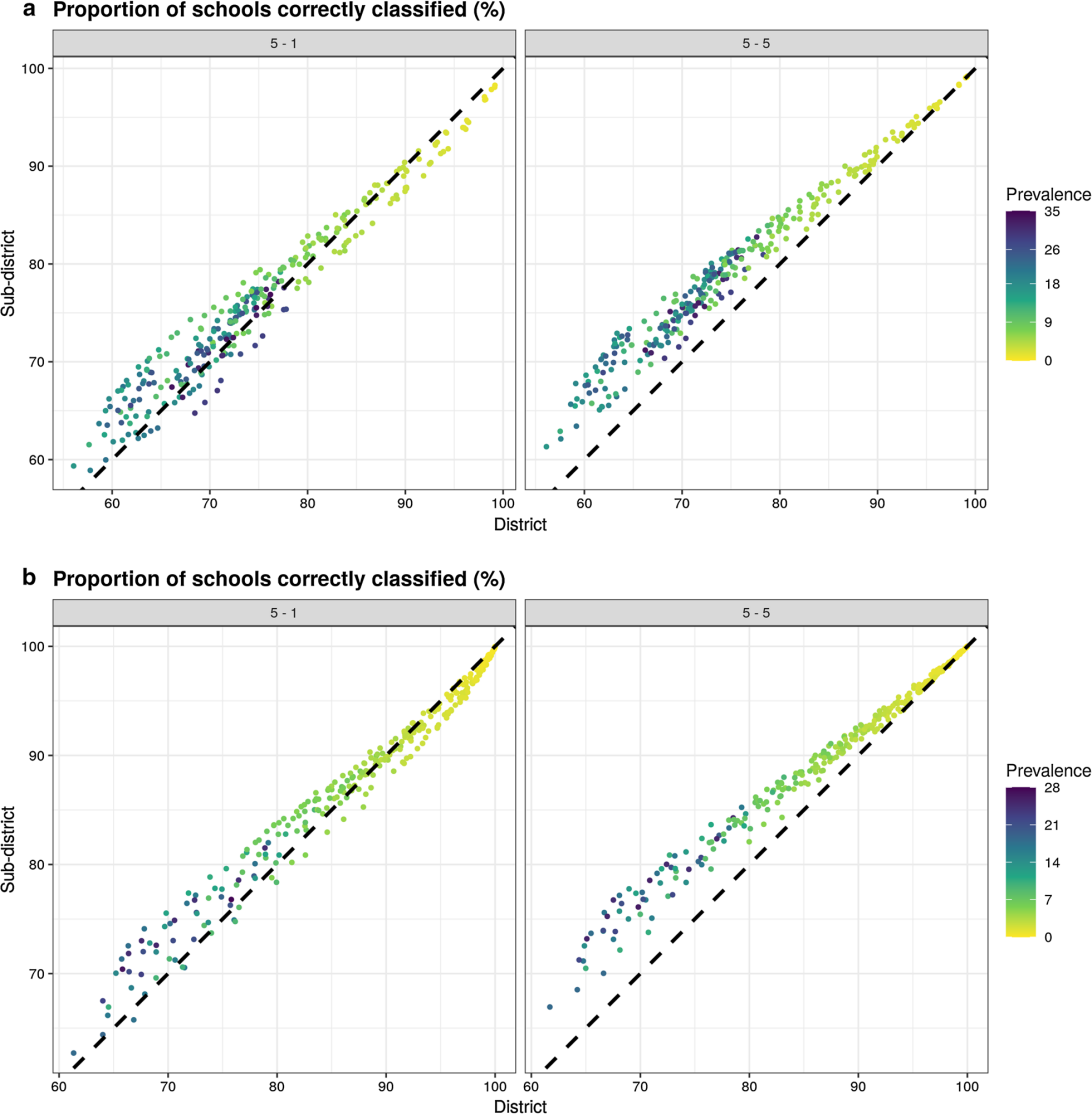
Comparisons of proportions of schools correctly classified sampling 5 schools per district *vs* 1 or 5 schools per subdistrict for *S. haematobium* (**a**) and *S. mansoni* (**b**)

Across these scenarios, the currently used protocol randomly sampling 5 schools with the IU defined at district level correctly classified a median of 86.7% (IQR: 83.4–90.3%) of districts for *S. haematobium* and 93.9% (IQR: 89.1–98.2%) of districts for *S. mansoni*. However, within these districts, only a median of 73.4% (IQR: 68.0–81.4%) and 89.2% (IQR: 80.1–96.3%) of schools were assigned to the correct treatment category for *S. haematobium* and *S. mansoni*, respectively. In contrast, defining the IU at subdistrict level and sampling 5 schools per IU correctly classified a slightly higher proportion of IUs, with a median of 89.8% (IQR: 86.9–92.1%) and 89.6% (IQR: 87.2–91.1%) subdistricts correctly classified for *S. haematobium* and *S. mansoni*. Similarly, within classified subdistricts, a higher proportion of schools were correctly classified, with 78.7% (IQR: 73.3–84.7%) and 75.7% (IQR: 70.9–79.3%) assigned the correct treatment category for *S. haematobium* and *S. mansoni* (Fig. 5).

**Fig. 6:**
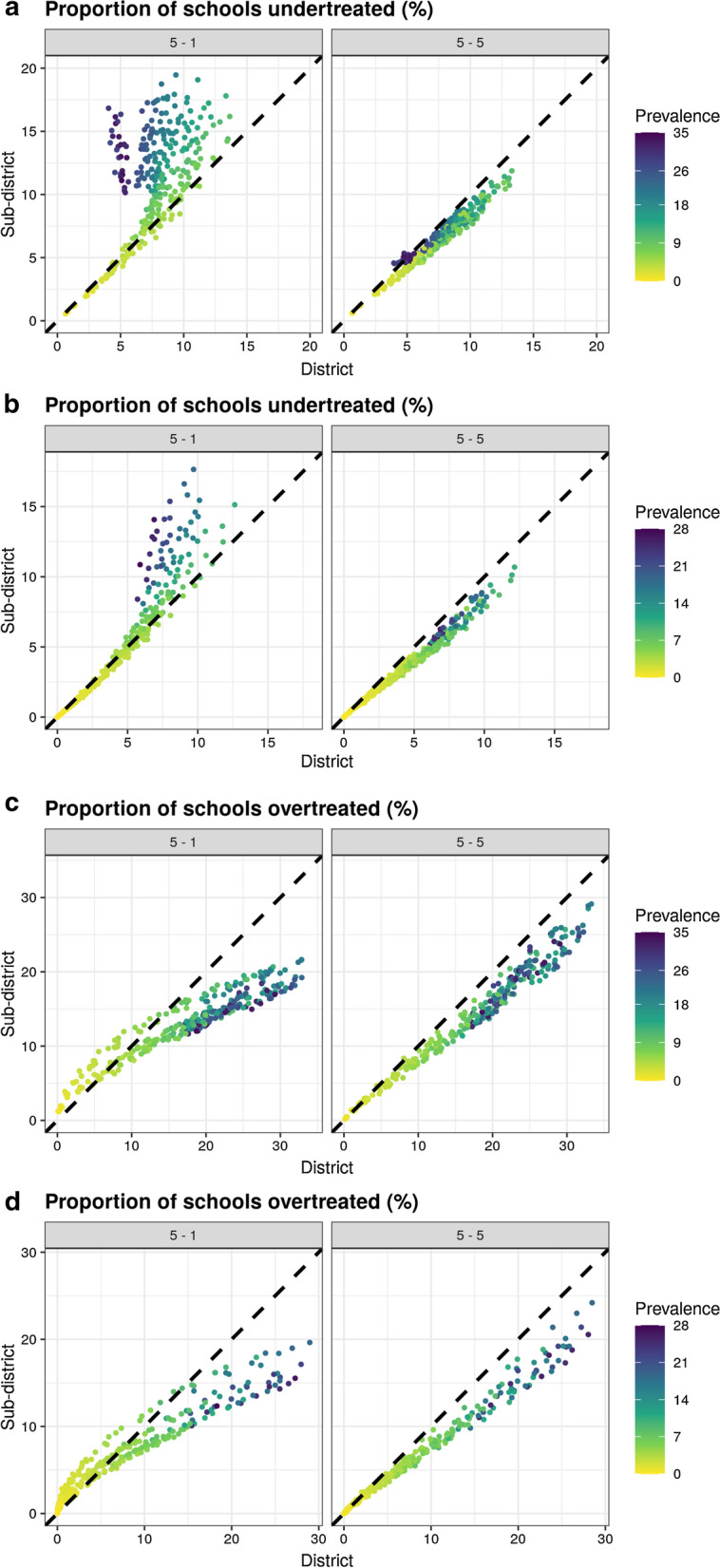
Comparisons of numbers of undertreated schools sampling 5 schools per per district *vs* 1 or 5 schools per subdistrict for *S. haematobium* (**a**) and *S. mansoni* (**b**) and numbers of overtreated schools for *S. haematobium* (**c**) and *S. mansoni* (**d**)

Due to the intensity of sampling required to survey 5 schools per subdistrict, we additionally evaluated a more realistic scenario sampling one school per subdistrict, sampling the same total number of schools as the current sampling approach of 5 schools per district. This slightly decreased the treatment classification accuracy for subdistrict level IUs, with the median probability of correctly classifying a subdistrict of 79.9% (IQR: 74.6–85.4%) and 91.7% (IQR: 85.7–96.6%) for *S. haematobium* and *S. mansoni* (Fig. 5). This led to treatment being correctly assigned to 75.2% (IQR: 69.5–82.2%) of schools for *S. haematobium* and 89.5% (IQR: 82.8–95.4%) of schools for *S. mansoni*. Comparing this sampling strategy to the current district level sampling strategy demonstrated that even sampling only one school per subdistrict still consistently resulted in a higher proportion of schools receiving the correct treatment for all but the lowest prevalence settings. Despite the overall accuracy, sampling more intensively at subdistrict level substantially decreased the number of undertreated schools when compared to sampling only one school per subdistrict (Fig. 6a, b). Any sampling strategy implemented at subdistrict level consistently decreased the number of overtreated schools (Fig. 6c, d). The treatment classification accuracy of all sampling strategies varied by schistosomiasis prevalence, with the lowest accuracy observed when prevalence approached the 10% threshold. Overall, increased focality (as represented by *φ*) resulted in increases in the numbers of schools over or undertreated for both species. Similarly, the accuracy of treatment classifications decreased with increasing spatial variance (*τ*^*2*^) and nonspatial variance (*σ*^*2*^). These effects were more pronounced when the mean prevalence approached the 10% threshold.

As the feasibility of these survey methods is largely dependent on total costs, we compared the total costs of each survey approach. Median estimated costs per school were USD 779.90 (IQR: USD 770.30–807.70) for schools surveyed with both urine filtration and Kato-Katz diagnostics, with median costs of USD 714.00 (IQR: USD 708.80–749.90) for schools surveyed using only urine filtration and USD 308.30 (IQR: USD 287.00–342.30) for schools surveyed using only Kato-Katz. In addition to being highly sensitive to the diagnostic method used, the majority of remaining costs were due to personnel costs (Additional file 1: Tables S2, S3). Although both sampling 5 schools per district and sampling 1 school per subdistrict resulted in the same number of schools and total costs within this scenario, the median costs per correctly classified school were slightly lower for both species when 1 school was sampled per subdistrict (Table 2). Sampling 5 schools per subdistrict resulted in the highest survey costs per correctly classified school. Similar trends were observed for costs per adequately treated school.

**Table 2:** Cost effectiveness of different sampling strategies for each species, median USD (IQR)

Species	Sampling strategy	Cost per correctly classified school	Cost per adequately treated school
*S. haematobium*	5 schools per district	22.71 (20.47–24.50)	25.26 (20.10–38.05)
	1 school per subdistrict	22.16 (20.27–23.96)	28.65 (23.72–37.42)
	5 schools per subdistrict	105.82 (98.40–113.63)	116.95 (98.19–153.03)
*S. mansoni*	5 schools per district	8.07 (7.47–8.98)	17.93 (12.62–39.92)
	1 school per subdistrict	8.04 (7.54–8.69)	16.36 (12.72–23.80)
	5 schools per subdistrict	39.32 (37.19–42.30)	67.73 (54.10–103.62)

## Discussion

Identifying and targeting mass drug administration to the most needed areas while minimising costs is a key priority for schistosomiasis control programmes. This study demonstrates the trade-offs control programmes are faced with when conducting mapping surveys to determine schistosomiasis treatment. A wide range of spatial patterns of both schistosomiasis species were identified across SSA, as expected, due to the diverse environments and health programmes within this region. For all of the varied schistosomiasis distributions assessed, using subdistrict level IUs consistently increases accuracy of both the IU level classification and the proportions of schools assigned to the correct treatment category. This analysis additionally illustrates how substantial proportions of schools may be undertreated, even when the IU is correctly classified, highlighting how the spatial structure impacts school-level classification accuracy. However, depending on the priorities of control programmes, the increased accuracy of more detailed mapping strategies will need to be weighed against the survey costs and whether the goals of the programme are to accurately distribute treatment or to minimise undertreatment.

Results of geostatistical analyses are consistent with other literature, reporting highly focal spatial distributions of schistosomiasis with large decreases in prevalence observed following periods of sustained control [2, 3]. Across all countries analysed, 6 countries had a mean school-level *S. haematobium* prevalence of 10% or greater and only 2 countries had a mean *S. mansoni* prevalence above this threshold. Previous studies have highlighted the dramatic decrease in schistosomiasis due to effective control programmes across this region and successes of preventive chemotherapy in substantially reducing disease burdens [4, 5]. However, due to limitations within the datasets analysed, changes over time could not be assessed for these data and there remains little understanding of how spatial distributions of schistosomiasis change following implementation of these control measures. While it is likely that patchy implementation and variable transmission intensity within existing implementation units will lead to increased focality, mapping surveys need to be evaluated against all plausible spatial distributions of schistosomiasis.

As would be expected, the spatial structure of schistosomiasis prevalence strongly influences the accuracy of treatment classifications under different mapping strategies. Classification accuracy decreases overall with increasing focality and spatial variance; these effects are particularly pronounced as mean prevalence approaches the 10% threshold. However, within these IU-level classifications, there is substantial heterogeneity in school-level prevalence, with the potential for large portions of schools to be over or undertreated despite correct classification of IU-level prevalence. Previous studies have similarly described substantial numbers of schools not assigned to the correct treatment categories under existing mapping strategies [16, 19]. This emphasises the need for control programmes to identify their key treatment priorities; in some cases, this may require adopting less accurate mapping strategies in favour of minimising the numbers of undertreated schools, prioritising sensitivity over accuracy.

Equally critically, control programmes are faced with choices on how to rationalise scarce resources. Within all survey designs considered, increasing the total numbers of schools surveyed consistently increases the classification accuracy. While we present the total costs per school surveyed and the cost per correctly identified school, cost effectiveness of different survey designs may vary substantially by country and diagnostic method. By comparing these survey costs with treatment costs for a particular region and species, control programmes can identify breakpoints at which the cost of treating all individuals within an IU for the next 5–6 years recommended by current guidelines is equal to or less than the costs of mapping [6]. For example, if a district has 100,000 SAC and treatment costs of USD 0.10 per child, the costs of treating all children annually over a 5-year period would be USD 50,000. Comparing these costs to the estimated survey costs allows control programmes to evaluate whether more intensive surveys would be more cost effective than treatment of all children within the IU. As treatment costs vary substantially by country and region, we have not evaluated survey cost effectiveness relative to treatment costs but instead provide a framework control programmes can use to evaluate these costs within specific settings. Detailed guidelines have been published for estimating both costs of surveys and treatments (e.g. [20, 21]), allowing control programmes to develop site specific budgets enabling planning of further survey activities. This may include evaluating economies of scale when rolling out large-scale mapping and treatment surveys [22]. This study highlights the importance of considering the spatial distributions of risk when designing surveys and demonstrates how simulations can allow assessment across a wide range of prevalence distributions.

Within these simulations, we chose to analyse the most commonly used two-stage cluster-based survey designs. This analysis assumed that the spatial distribution of prevalence is unknown with no previously available geolocated school survey data, optimising sampling under a range of prevalence levels and spatial distributions. While most countries have some baseline survey data, many countries lack reliable spatial information on prevalence. However, if previous spatial data are available, alternative sampling strategies may be more cost effective and appropriate. For example, geostatistical sampling designs utilise information on the spatial structure of disease prevalence to design more efficient surveys [23]. Exploiting spatial correlation between locations, this approach uses spatially regulated sampling within a model-based geostatistical framework to estimate prevalence surfaces. While we only consider methods for classifying an IU based on the empirical mean as typically done by current practice, geostatistical analysis with a probabilistic assessment of the IU endemicity would lead to a substantial improvement of classification accuracy. Alternatively, when previous data are available, adaptive sampling can be used to identify disease hotspots, applying previous survey information to optimise sampling strategies [24]. While these sampling approaches are likely to be more efficient than simple random sampling, our analysis provides a framework for collecting baseline prevalence data with little or no previous geolocated survey data or in countries without the resources or technical skills to implement more complex statistical models

Despite the utility of this approach in evaluating sampling strategies, this study had several important limitations. Due to the wide range of spatial patterns assessed, we chose to conduct unconditional simulations across a simulated country. While this allows assessment of spatial distributions of risk representative of those found across SSA, the parameters used do not reflect the density of schools or distributions of administrative districts of all countries. Subdistrict definitions vary substantially by country, with notable increases in the numbers of subnational administrative units within the past decades [25]. While the consistent numbers of subdistricts identified in all districts within our simulated country are unlikely to occur in most countries, this provides a model of how control programmes could consider subdividing districts with variable numbers of subdistricts. Further, as this modelling approach was developed using open-source software, this analysis can be easily modified to reflect actual administrative boundaries. Additionally, although this study analysed the largest available database of school-based surveys for schistosomiasis, this did not allow the evaluation of how spatial patterns changed over time or in response to control measures. Future studies could evaluate how these spatial distributions would be expected to change. As surveys are only conducted in geographically suitable regions for schistosomiasis transmission, further work could explore the application of improved environmental data in defining and identifying these regions.

## Conclusions

Together, this study demonstrates the importance of spatial structure when designing mapping surveys and highlights how using finer scale implementation units can improve treatment classification accuracy. While the most substantial gains in accuracy result from sampling higher numbers of schools at subdistrict levels, even sampling the same number of schools stratified by subdistrict improves overall accuracy. Effects of these survey designs become more pronounced as focality and variance increases and prevalence approaches the 10% threshold. By considering the specific goals of the control programme, costs and likely spatial distributions within the study areas, this analysis can be used to guide control programmes to develop appropriate sampling strategies.

## Supplementary information

**Additional file 1. Figure S1.** Distribution of prevalence from sampled schools. **Table S1.** Monte Carlo maximum likelihood estimates for geostatistical model parameters for S. haematobium (a) and S. mansoni (b). **Figure S2.** Simulated country and distributions of districts, subdistricts and schools. **Table S2. **Consumable and equipment costs included for sampling 50 children using urine filtration (A) and Kato-Katz stool analysis (B); life expectancy and quantity needed per team is included for capital items. **Table S3. **Total survey mapping costs per school sampling 50 children. **Table S4. **Mean survey costs per school sampling 50 children by country, year and diagnostic method. (DOCX 950 kb)

## Data Availability

All data and R source code needed to replicate this analysis are publicly available at: https://github.com/claudiofronterre/schisto_paper_KF_CF.

## Abbreviations

SSA: Sub-Saharan Africa
IU: Implementation unit
