# Supplementary material for: Evaluating survey designs for targeting preventive chemotherapy against Schistosoma haematobium and Schistosoma mansoni across sub-Saharan Africa: a geostatistical analysis and modelling study
Source: Parasit Vectors. 2020 Nov 18;13:555. doi: 10.1186/s13071-020-04413-7 (PMC7672864; doi:10.1186/s13071-020-04413-7)
Supplement: Supplementary file 1 — Additional file 1. Figure S1. Distribution of prevalence from sampled schools. Table S1. Monte Carlo maximum likelihood estimates for geostatistical model parameters for S. haematobium (a) and S. mansoni (b). Figure S2. Simulated country and distributions of districts, subdistricts and schools. Table S2. Consumable and equipment costs included for sampling 50 children using urine filtration (A) and Kato-Katz stool analysis (B); life expectancy and quantity needed per team is included for capital items. Table S3. Total survey mapping costs per school sampling 50 children. Table S4. Mean survey costs per school sampling 50 children by country, year and diagnostic method. (DOCX 950 kb) [file 13071_2020_4413_MOESM1_ESM.docx]

**Additional file 1**

**Supplementary Information.** Evaluating survey designs for targeting preventive chemotherapy against *Schistosoma haematobium* and *S. mansoni* across Sub-Saharan Africa: a geostatistical analysis and modelling study

**Figure S1.** Distribution of prevalence from sampled schools


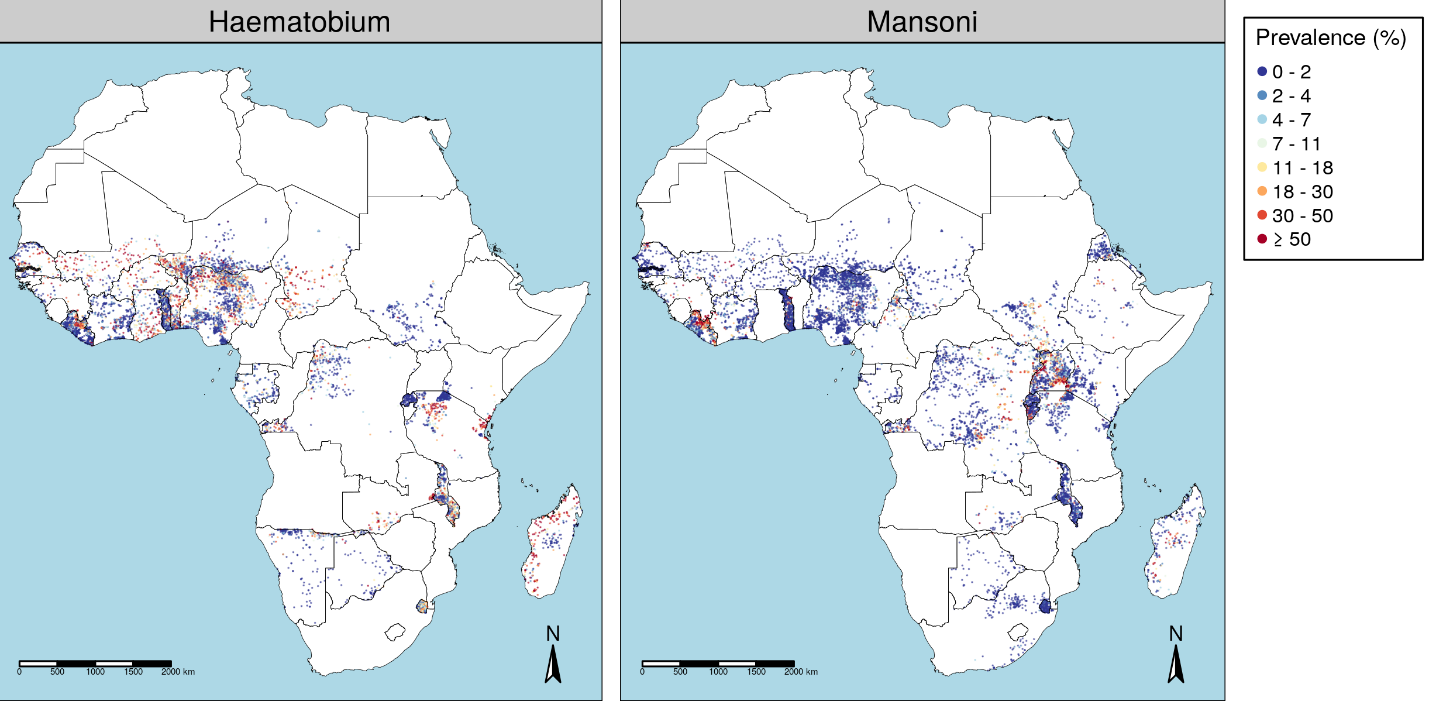


**Table S1.** Monte Carlo maximum likelihood estimates for geostatistical model parameters for A) *S. haematobium* and B) *S. mansoni*

A.

| Country | n | *µ* | *σ^2^* | *φ* | *τ^2^* |
| --- | --- | --- | --- | --- | --- |
| Benin | 358 | 0.088 | 3.386 | 18.199 | 1.872 |
| Botswana | 93 | 0.013 | 0.307 | 28.206 | 1.827 |
| Burkina Faso | 101 | 0.119 | 3.621 | 8.826 | 0.000 |
| Chad | 241 | 0.108 | 3.663 | 79.820 | 0.857 |
| Congo (Kinshasa) | 387 | 0.015 | 4.185 | 78.600 | 2.580 |
| Cote D'Ivoire | 476 | 0.006 | 5.188 | 60.096 | 1.164 |
| Gabon | 199 | 0.011 | 1.484 | 89.428 | 0.632 |
| Ghana | 166 | 0.079 | 4.036 | 110.629 | 1.695 |
| Guinea | 64 | 0.053 | 10.428 | 69.858 | 1.825 |
| Kenya | 104 | 0.055 | 11.519 | 31.712 | 4.489E^-4^ |
| Liberia | 1238 | 0.010 | 6.751 | 90.541 | 0.814 |
| Madagascar | 259 | 0.140 | 6.924 | 135.904 | 1.097 |
| Malawi | 858 | 0.046 | 1.331 | 76.750 | 1.615 |
| Mali | 164 | 0.128 | 4.900 | 91.911 | 1.647 |
| Niger | 825 | 0.021 | 1.891 | 138.990 | 5.138 |
| Nigeria | 1838 | 0.035 | 3.736 | 87.865 | 1.880 |
| Rwanda | 158 | 0.007 | 0.005 | 5.179 | 0.001 |
| Senegal | 218 | 0.022 | 5.629 | 86.692 | 3.927 |
| South Sudan | 225 | 0.004 | 1.380 | 44.596 | 0.463 |
| Swaziland | 251 | 0.056 | 1.673 | 41.355 | 0.520 |
| Tanzania (Mainland) | 587 | 0.033 | 10.075 | 146.441 | 0.571 |
| The Gambia | 195 | 0.108 | 0.804 | 0.074 | 2.795E^-12^ |
| Togo | 931 | 0.017 | 2.416 | 31.370 | 0.615 |
| Zambia | 92 | 0.207 | 1.094 | 19.365 | 0.842 |

B.

| Country | n | *µ* | *σ^2^* | *φ* | *τ^2^* |
| --- | --- | --- | --- | --- | --- |
| Botswana | 111 | 0.001 | 1.910 | 372.268 | 0.346 |
| Burkina Faso | 89 | 0.004 | 1.094 | 110.351 | 1.00E^-13^ |
| Burundi | 246 | 0.020 | 7.264 | 45.854 | 5.341 |
| Cameroon | 116 | 0.047 | 3.040 | 10.800 | 8.70E^-12^ |
| Chad | 232 | 0.003 | 3.941 | 696.132 | 0.423 |
| Congo (Kinshasa) | 1391 | 0.007 | 8.377 | 163.319 | 1.316 |
| Cote D'Ivoire | 479 | 0.014 | 4.876 | 106.220 | 1.086 |
| Eritrea | 329 | 0.003 | 1.868 | 56.097 | 1.066 |
| Ethiopia | 65 | 0.007 | 6.034 | 249.102 | 5.525 |
| Gabon | 86 | 0.005 | 0.294 | 7.118 | 5.75E^-12^ |
| Guinea | 64 | 0.099 | 6.989 | 85.362 | 1.943 |
| Kenya | 788 | 0.001 | 13.183 | 47.460 | 0.593 |
| Liberia | 1193 | 0.066 | 4.352 | 58.822 | 0.570 |
| Madagascar | 226 | 0.008 | 7.898 | 55.156 | 1.196 |
| Malawi | 857 | 0.007 | 0.891 | 44.406 | 1.250 |
| Mali | 182 | 0.001 | 9.250 | 87.455 | 2.202 |
| Niger | 358 | 0.004 | 0.389 | 64.458 | 0.646 |
| Nigeria | 2230 | 0.004 | 1.851 | 88.454 | 0.424 |
| Rwanda | 158 | 0.004 | 2.840 | 9.615 | 6.03E^-14^ |
| Senegal | 221 | 0.000 | 9.277 | 233.162 | 0.883 |
| South Africa | 180 | 0.002 | 0.006 | 0.005 | 0.209 |
| South Sudan | 393 | 0.006 | 8.251 | 164.494 | 0.663 |
| Swaziland | 251 | 0.006 | 0.120 | 1.187 | 3.97E^-12^ |
| Tanzania (Mainland) | 553 | 0.005 | 4.171 | 88.247 | 0.781 |
| The Gambia | 222 | 0.008 | 0.023 | 4.941 | 0.032 |
| Togo | 1052 | 0.007 | 3.355 | 33.755 | 0.382 |
| Uganda | 1233 | 0.040 | 4.723 | 49.332 | 1.652 |
| Zambia | 101 | 0.029 | 5.407 | 124.995 | 1.955 |

**Figure S2.** Simulated country and distributions of districts, subdistricts and schools


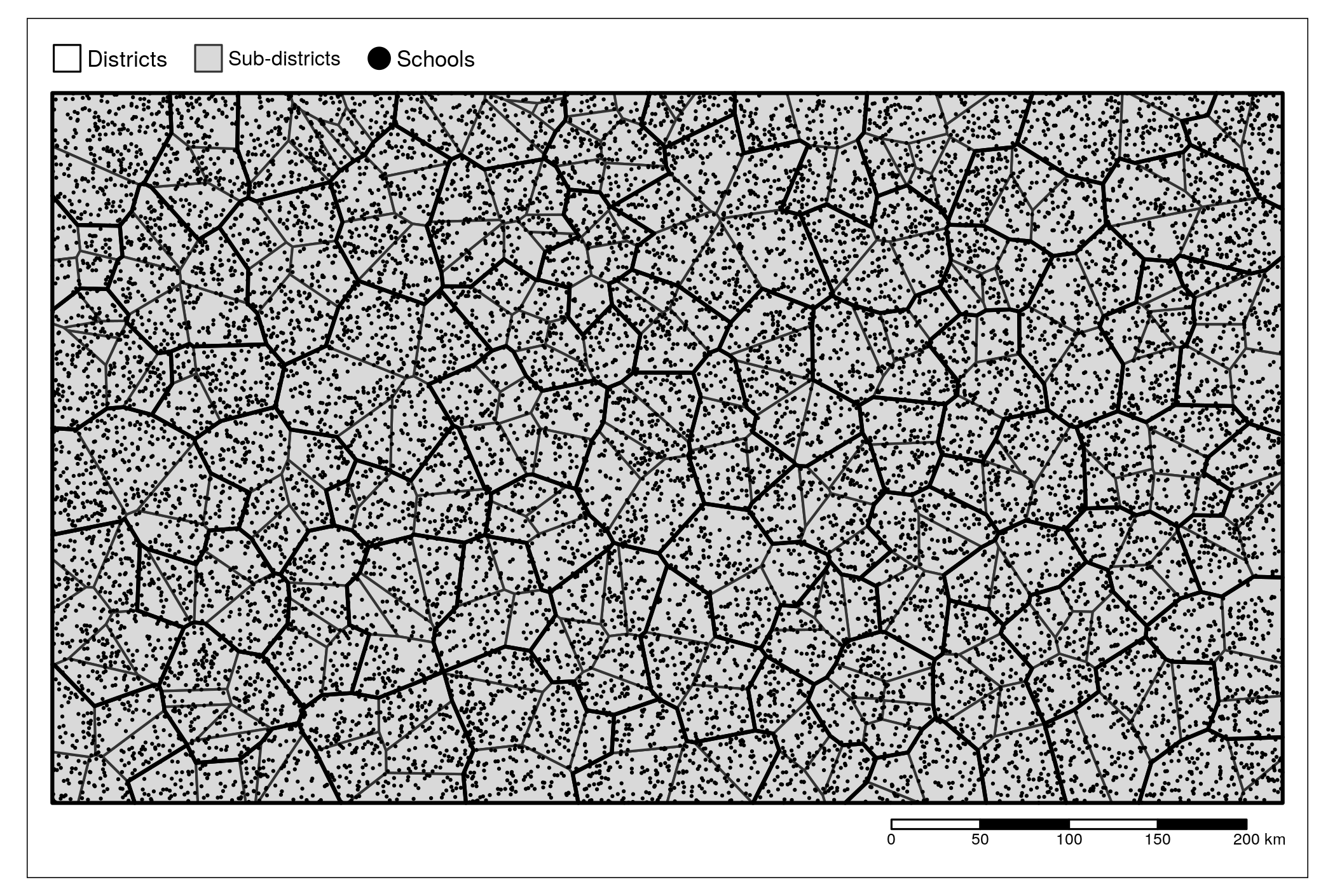


**Table S2.** Consumable and equipment costs included for sampling 50 children using A) urine filtration and B) Kato Katz stool analysis; life expectancy and quantity needed per team is included for capital items

A.

| Item | | Quantity per school | Life expectancy (years) |
| --- | --- | --- | --- |
| Consumables | | | |
|  | Nucleopore membrane filter (13mm and pore size 12µm) | 50 |  |
|  | Masking tape | 2.5 rolls |  |
|  | Detergent | 100g |  |
|  | Bleach (1% hypochlorite solution) | 50ml |  |
|  | Gloves | 0.2 boxes |  |
|  | Toilet tissue | 2.5 rolls |  |
|  | Hemostix reagent strips | 50 |  |
|  | Lugol’s iodine (5% solution) | 20ml |  |
|  | Slide boxes | 0.2 box |  |
| Capital items | |  |  |
|  | Filter holders | 60 | 4 |
|  | Gaskets | 60 | 4 |
|  | Microscopes with removable mirrors | 5 | 5 |
|  | Pencils | 9 | 2 |
|  | Pens | 9 | 2 |
|  | Fine tip permanent marker | 1 | 2 |
|  | Notepad | 3 | 2 |
|  | Old newspapers | 5kg | 2 |
|  | Scissors | 1 | 5 |
|  | Large plastic buckets with lids | 1 | 5 |
|  | Rubber washing gloves | 1 pair | 2 |
|  | Generator (powers up to 6 microscopes) | 1 | 5 |
|  | Urine sample pots (20ml) | 60 pots | 5 |
|  | Forceps/ tweezers | 1 | 5 |
|  | Pipettes | 1 | 2 |
|  | Syringe (10ml) | 1 | 2 |
|  | Hand tally counter | 1 | 5 |
|  | Microscopy glass slides | 60 | 5 |
| Additional costs* | |  |  |

* Calculated as 5% of additional survey costs including purchase of packing containers, power extension cable, large plastic bowls, small plastic bowls with lids, water container, toothbrushes, water brushes, antibacterial hand wash, hand sanitiser, disposable plastic aprons, cotton towels, kitchen roll, saline solution (0.9% NaCl), distilled water, Malachite green (or methylene blue), glycerol, wooden applicator, slide labels, and tissue for microscope

B.

| Item | | Quantity per school | Life expectancy (years) |
| --- | --- | --- | --- |
| Consumables | | | |
|  | Cellophane (sheets) | 2.1 sheets |  |
|  | Masking tape | 2.5 rolls |  |
|  | Detergent | 100g |  |
|  | Bleach (1% hypochlorite solution) | 50ml |  |
|  | Gloves | 0.2 box |  |
|  | Toilet tissue | 2.5 rolls |  |
|  | Stool sample container (polyethene squares) | 0.02m^2^ |  |
|  | Slide boxes (for 10% of slides) | 0.45 box |  |
|  | Insecticide spray | 0.1 bottle |  |
|  | Biohazard waste bins | 1 bin |  |
| Capital items | |  |  |
|  | Metal sieve (Endocott sieve, 200-250µm mesh size) | 1 sieve | 3 |
|  | Kato Katz plastic templates (6mm hole and 1.5mm thick, 41.7mg) | 0.5 box | 4 |
|  | Microscopes with removable mirrors | 3 | 5 |
|  | Pencils | 9 | 2 |
|  | Pens | 9 | 2 |
|  | Fine tip permanent marker | 9 | 2 |
|  | Notepad | 3 | 2 |
|  | Old newspapers | 5kg | 2 |
|  | Scissors | 1 | 5 |
|  | Large plastic buckets with lids | 1 | 5 |
|  | Rubber washing gloves | 1 pair | 2 |
|  | Generator (powers up to 6 microscopes) | 1 | 5 |
| Additional costs* | |  |  |

* Calculated as 5% of additional survey costs including purchase of packing containers, power extension cable, large plastic bowls, small plastic bowls with lids, water container, toothbrushes, water brushes, antibacterial hand wash, hand sanitiser, disposable plastic aprons, cotton towels, kitchen roll, saline solution (0.9% NaCl), distilled water, Malachite green (or methylene blue), glycerol, wooden applicator, slide labels, and tissue for microscope

**Table S3.** Total survey mapping costs per school sampling 50 children

| Item | Total cost in USD, median (IQR) |
| --- | --- |
| Personnel (per diems for survey staff) | 205.30 (201.90 – 226.60) |
| Transport (fuel and vehicle maintenance) | 46.65 (34.19 – 51.75) |
| Consumables and equipment – urine filtration | 465.40 (464.40 – 468.30) |
| Consumables and equipment – Kato Katz | 58.85 (57.85 – 60.66) |

**Table S4.** Mean survey costs per school sampling 50 children by country, year and diagnostic method

| Country | Survey Year | Diagnostic Method | Total cost in USD |
| --- | --- | --- | --- |
| Malawi | 2017 | Kato Katz and urine filtration | 807.71 |
| Uganda | 2017 | Kato Katz and urine filtration | 696.71 |
| Malawi | 2018 | Kato Katz and urine filtration | 828.18 |
| Tanzania | 2018 | Kato Katz and urine filtration | 779.92 |
| Uganda | 2018 | Kato Katz and urine filtration | 770.34 |
| Malawi | 2017 | Urine filtration | 749.86 |
| Uganda | 2017 | Urine filtration | 636.05 |
| Malawi | 2018 | Urine filtration | 769.33 |
| Tanzania | 2018 | Urine filtration | 708.84 |
| Uganda | 2018 | Urine filtration | 714.03 |
| Malawi | 2017 | Kato Katz | 342.27 |
| Uganda | 2017 | Kato Katz | 228.43 |
| Malawi | 2018 | Kato Katz | 363.77 |
| Tanzania | 2018 | Kato Katz | 286.99 |
| Uganda | 2018 | Kato Katz | 308.28 |
